# *Lysimachia huangsangensis* (Primulaceae), a New Species from Hunan, China

**DOI:** 10.1371/journal.pone.0132713

**Published:** 2015-07-22

**Authors:** Jian-Jun Zhou, Xun-Lin Yu, Yun-Fei Deng, Hai-Fei Yan, Zhe-Li Lin

**Affiliations:** 1 School of Forestry, Central South University of Forestry & Technology, 410004, Changsha, People’s Republic of China; 2 Key Laboratory of Plant Resources Conservation and Sustainable Utilization, South China Botanical Garden, the Chinese Academy of Sciences, Guangzhou 510650, People’s Republic of China; 3 University of Chinese Academy of Sciences, Beijing 100049, People’s Republic of China; University of Florida, UNITED STATES

## Abstract

A new species, *Lysimachia huangsangensis* (Primulaceae), from Hunan, China is described and illustrated. The new species is closely related to *L*. *carinata* because of the crested calyx, but differs in the leaf blades that are ovate to elliptic and (3–)4.5–9 × 2–3.4 cm, 2–5-flowered racemes, and the calyx lobes that are ovate-lanceolate and 5–6 × 3–4 mm. The systematic placement and conservation status are also discussed.

## Introduction

*Lysimachia* L. belongs to the tribe Lysimachieae Reich. and consists of 140–200 species with an almost worldwide distribution but exhibits striking local endemism [1–7]. China is one of the centers of diversity for *Lysimachia*, being home to approximately 140 species [5, 8–20].

Traditionally *Lysimachia* was recognized as a primitive group in the family Primulaceae and to be related to the family Myrsinaceae [21–28]. Molecular phylogenetic studies as well as morphological data have supported the transfer of *Lysimachia* with other genera of the tribe Lysimachieae to the family Myrsinaceae [4, 29–34]. However, in the recent system of the Angiosperm Phylogeny Group [35–37], Mysinaceae is merged with Primulaceae and the Primulaceae are divided into four subfamilies: Maesoideae, Theophrastoideae, Myrsinoideae, and Primuloideae [38].

Many taxonomists have tried to establish an infrageneric classification for the genus. Klatt [39] was the first to subdivide the genus and recognized nine sections. Pax & Knuth [21]went on to divide the genus into 16 sections. Handel-Mazzetti [22] established a new arrangement for the genus and divided it into five subgenera, viz., subgen. *Lysimachia*, subgen. *Idiophyton* Hand.-Mazz., subgen. *Naumburgia* (Moench) Hand.-Mazz., subgen. *Lysimachiopsis* (Heller) Hand.-Mazz. and subgen. *Pallidia* (Moench) Hand.-Mazz. His system constitutes the most important contribution to the classification of *Lysimachia* and was followed by many authors with minor modifications [8, 9, 24, 34, 40]. Ray [24] separated species of the New World into five subgenera. Chen & Hu [8, 9] divided Chinese *Lysimachia* into five subgenera adding subgen. *Heterostylandra* (Hand.-Mazz.) F.H. Chen &C.M. Hu and subgen. *Lysimachiopsis*, which does not occur in China.

Recent phylogenetic analyses [32, 34, 41–44] found that the genus *Lysimachia* in the traditional sense was paraphyletic, and some related satellite genera, viz., *Anagallis* L., *Asterolinon* Hoffmans. et Link, *Glaux* L. and *Pelletiera* A. St.-Hil., were nested within *Lysimachia*. Accordingly, *Lysimachia* was expanded to include these satellite genera to maintain *Lysimachia* as a monophyletic entity [45, 46]. The traditional subgeneric classification was re-evaluated by Anderberg *et al*.[34].

In the course of field work in SW Hunan Province, China, a distinct species of *Lysimachia* with a crested calyx was found. Further studies showed that it represented a new species belonging to ser. *Drymarifoliae* Hand.-Mazz. in subgen. *Lysimachia sensu* Chen & Hu [8]. Ser. *Drymarifoliae* includes about 10 species in China and is characterized by having the flowers arranged in umbel-like, axillary, 1–several-flowered racemes and filaments connate at the base [9]. In this series, the species can be divided into two groups according to whether the calyx has crested ridges or not. The new species belongs to the group having a crested calyx.

## Material and Methods

### Ethics statement

The new species reported in this work was collected from Huangsang Natural Reserve and Yunshan Natural Reserve, Hunan, China, which are protected by the Forestry Bureau of Hunan Province. The Forestry Bureau permits research in these reserves and no specific permits are required for the present study. The field studies did not involve endangered or protected species.

### Morphological observations

The morphological description of the new species was based on the examination of fresh and pressed specimens. The morphological comparison with related species, *L*. *carinata* Y. I. Fang & C. Z. Zheng, *L*. *pterantha* Hemsl., *L*. *baoxingensis* (F. H. Chen &C. M. Hu) C. M. Hu, *L*. *pteranthoides* Bonati and *L*. *crista-galli* Pamp. ex Hand.-Mazz., was based on studies of herbarium specimens and information gathered from literature searches. The specimens examined were deposited in the following herbaria: CDBI, IBSC, K, NAS, P, PE and WUK. The herbarium acronyms follow the Index Herbariorum [47].

The pollen grains and seeds were directly mounted on aluminium stubs coated with gold in a sputter coater and examined using scanning electron microcopy (SEM). The polar (P) axis and equatorial (E) diameter were measured by imaging analyzer (Smile View 2.1; JEOL Tokyo, Japan). Pollen terminology follows Erdtman [48] and Bennell & Hu [40]. Seed terminology follows Oh *et al*.[28].

### Nomenclature

The electronic version of this article in Portable Document Format (PDF) in a work with an ISSN or ISBN will represent a published work according to the International Code of Nomenclature for algae, fungi, and plants, and hence the new names contained in the electronic publication of a PLOS article are effectively published under that Code from the electronic edition alone, so there is no longer any need to provide printed copies.

In addition, new names contained in this work have been submitted to IPNI, from where they will be made available to the Global Names Index. The IPNI LSIDs can be resolved and the associated information viewed through any standard web browser by appending the LSID contained in this publication to the prefix http://ipni.org/. The online version of this work is archived and available from the following digital repositories: PubMed Central and LOCKSS.

### Taxon sampling

In order to determine the systematic position of the new species, we proceeded to create a phylogenetic analysis by adding published sequences of Chinese *Lysimachia* species. We retrieved a total of 66 sequences for 25 species downloading those published in a barcoding study of Chinese *Lysimachia* [16] from GenBank, In addition, three samples representing the new species were included in this study.

### Molecular markers

Total genomic DNA was extracted from silica-dried plant leaves using a modified CTAB protocol [49], and then target DNA regions including the two core barcodes (*rbcL* and *matK*), *trnH-psbA* and ITS were amplified with common DNA barcoding primers. Primer pairs, PCR amplification and sequencing conditions following Zhang *et al*. [16] and Chen *et al*. [50]. GenBank accession numbers for all the DNA sequences and voucher information are given in S1 Table.

### Sequence alignment and phylogenetic analysis

Sequence alignment was initially performed using with MUSCLE 3.8.31 [51] in the multiple alignment routine followed by manual adjustment in Se-Al v2.0a11 (http://tree.bio.ed.ac.uk/software/seal/). Given that the plastid genome behaves as a single linked region, the three plastid markers (*rbc*L, *mat*K and *trn*H-*psb*A) were concatenated *a priori*. Congruence between the combined plastid fragments and nuclear marker ITS was tested with the incongruence length difference (ILD) test [52], which was conducted using PAUP* version 4.0b10with 100 replicates of the heuristic search (default setting) [53]. The 1% level of significance was chosen as described in [54]. The ILD value in this study was 0.02 and we therefore,decide to combined all the datasets (*rbc*L, *mat*K,*psb*A*-trn*H, and ITS) in our analysis using Maximum Likelihood (ML) analysis. We first determined the best-fitting model of sequence evolution for the total data matrix using ModelTest 3.7 [55]. Results of the Akaike information criterion (AIC, [56]) indicated that the TIM + I + G model was the best-fitting model for the combined data matrix. The ML analysis was performed using GARLI Web Service [57, 58] with the best-fitting model (http://www.molecularevolution.org/software/phylogenetics/garli). Default parameters were used in this analysis, and 10 independent search replicates were run with each replicate run. Bootstrap support values for nodes on the ML topology were computed with GARLI by running 500 bootstrap replicates.

Bayesian inference (BI) was conducted using MrBayes version 3.2.1 [59] with the best-fitting model (TIM + I + G). The Bayesian Markov chain Monte Carlo (MCMC) algorithm was run for 5000000 generations with four incrementally heated chains starting from random trees and sampling one out of every 1000 generations. A conservative burn-in (25%) was applied after checking for stability on the log-likelihood curves and split variances less than 0.01. A majority rule consensus tree was calculated from the remaining trees. The internodes support was determined by Bayesian Posterior Probabilities.

## Results

### Taxonomic treatment

#### Lysimachia huangsangensis

**Fig 1:**
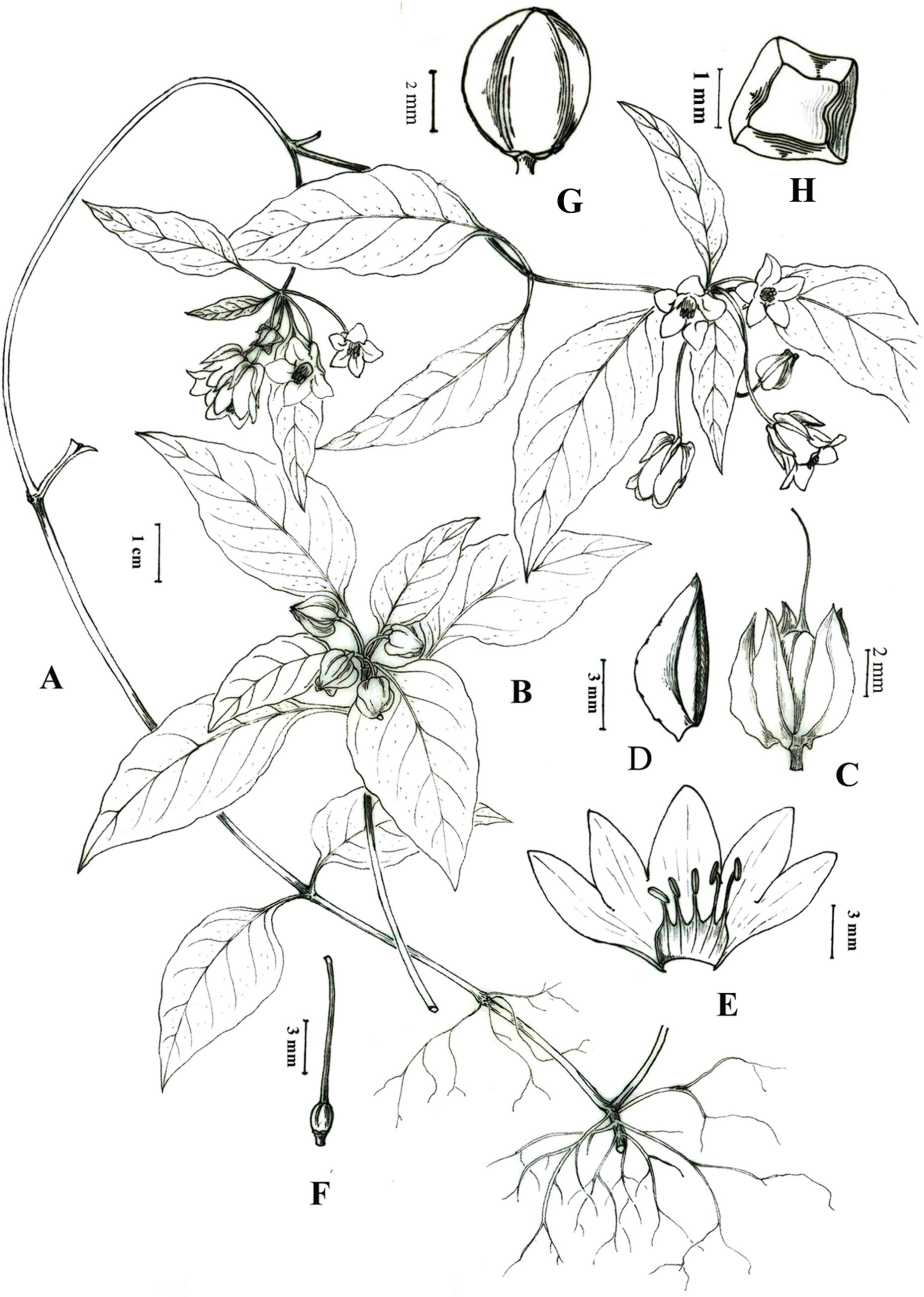
*Lysimachia huangsangensis*. A. Habit; B. Fruiting branch; C. Calyx; D. Calyx-lobe showing crest ridge; E. Open corolla with stamens; F. Ovary and style; G. Fruit; H. seed. Drawn by Jing TIAN. (A-F from *J*. *J*. *Zhou & D*. *Zhou 13338*, G from *J*. *J*. *Zhou 14101902*).

**Fig 2:**
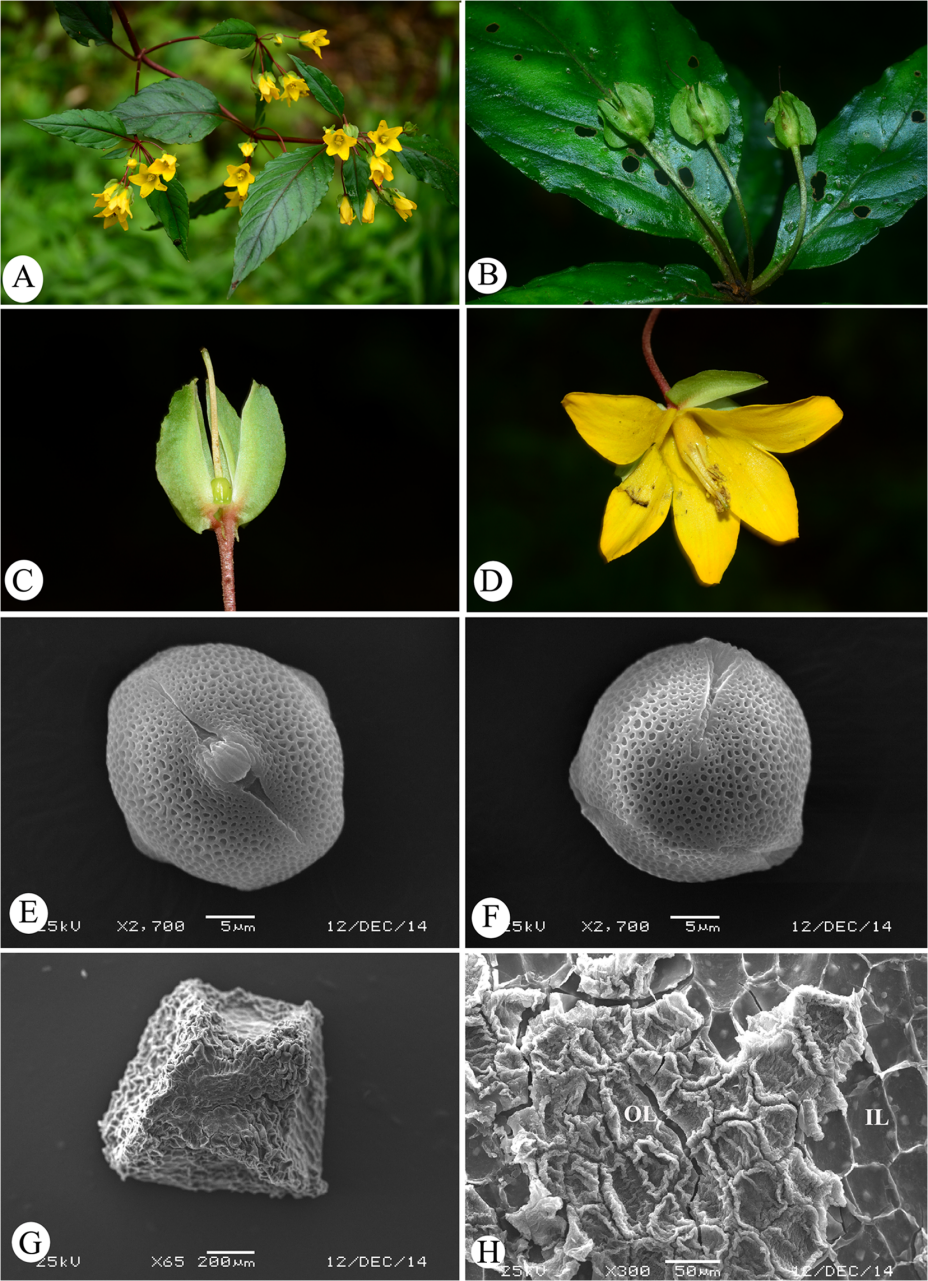
Lysimachia huangsangensis. A. Flowering branch; B. Fruiting branch; C. Calyx and pistile; D. Flower; E. Equatorial view of pollen grain; F. Polar view of pollen grain; G. Seed; H. Ornamentation of seed surface. IL inner layer of the seed coat; OL outer layer of the seed coat.

[urn:lsid:ipni.org:names:77147698–1] J.J. Zhou, X.L. Yu & Y.F. Deng, sp. nov. (Figs 1 and 2). Type: China. Hunan: Suiningxian, Huangsang Natural Reserve, Anyangshan Cun, ca. 480 m, 20 May 2013, *J*. *J*. *Zhou &D*. *Zhou 13338* (holotype CSFI; isotype IBSC).

### Diagnosis

The new species is similar to *Lysimachia carinata* Y.I. Fang & C.Z. Zheng, but differs in ovate to elliptic and (3–)4.5–9 × 2–3.4 cm (versus broadly ovate to ovate and 1.5–2.5 × 1–2 cm), the 2–5-flowered racemes (versus 1–2-flowered) and the calyx lobes that are ovate-lanceolate and 5–6 ×3–4 mm (versus linear-lanceolate and ca. 4 × 1 mm).

### Description

Perennial herbs, to 40 cm tall. Stems purplish-red, procumbent at base, then erect, simple or rarely branched from base, glabrous. Leaves opposite; petioles 0.5–2 cm, purplish-red, glabrous, narrowly winged toward the base; blades ovate to elliptic, (3–)4.5–9 × 2–3.4 cm, abaxially glabrous, adaxially sparsely bristly, base rounded to narrowly cuneate, decurrent onto the petiole, margin entire to slightly undulate, apex acute to acuminate, midvein purplish, slightly impressed adaxially, prominent abaxially, lateral veins 6–8 pairs, flat adaxially, prominent abaxially, tertiary veins inconspicuous. Raceme axillary, 2–5-flowered; peduncle and rachis short, 1–3 mm long; pedicel slender, purplish-red, 1.5–3.5 cm long, glabrous. Calyx 5-lobed to the base, lobes ovate, 5–6 × 3–4mm, glabrous, abaxially crested, base rounded, margin entire, apex acute; crest 1–2.5 mm, wing-like, widest at the base, glabrous. Corolla 1–1.2 cm, yellow, glabrous, tube 5–6 mm, lobes ovate to elliptic, 5–7 × 4–5 mm, apex acute, margin entire. Stamens 5, yellow, 6–8 mm long, base connate into a tube for 1/3–1/2; anthers yellow, oblong, ca. 1.5 mm, dorsifixed, opening by lateral splits; pollen grains medium-sized, subprolate, 29.39 (26.3–34.3) ×25.31 (23.2–27.1) μm, tricolporate, P/E = 1.36, and tectum reticulate. Ovary ovoid, ca. 1.5 mm, glabrous; styles slightly longer than the stamens, glabrous. Capsule subspherical, ca. 5 mm in diameter, glabrous. Seeds polyhedral with lateral faces slandered towards the ventral face, black, ca. 1 mm, surface reticulate.

### Distribution and habitat

**Fig 3:**
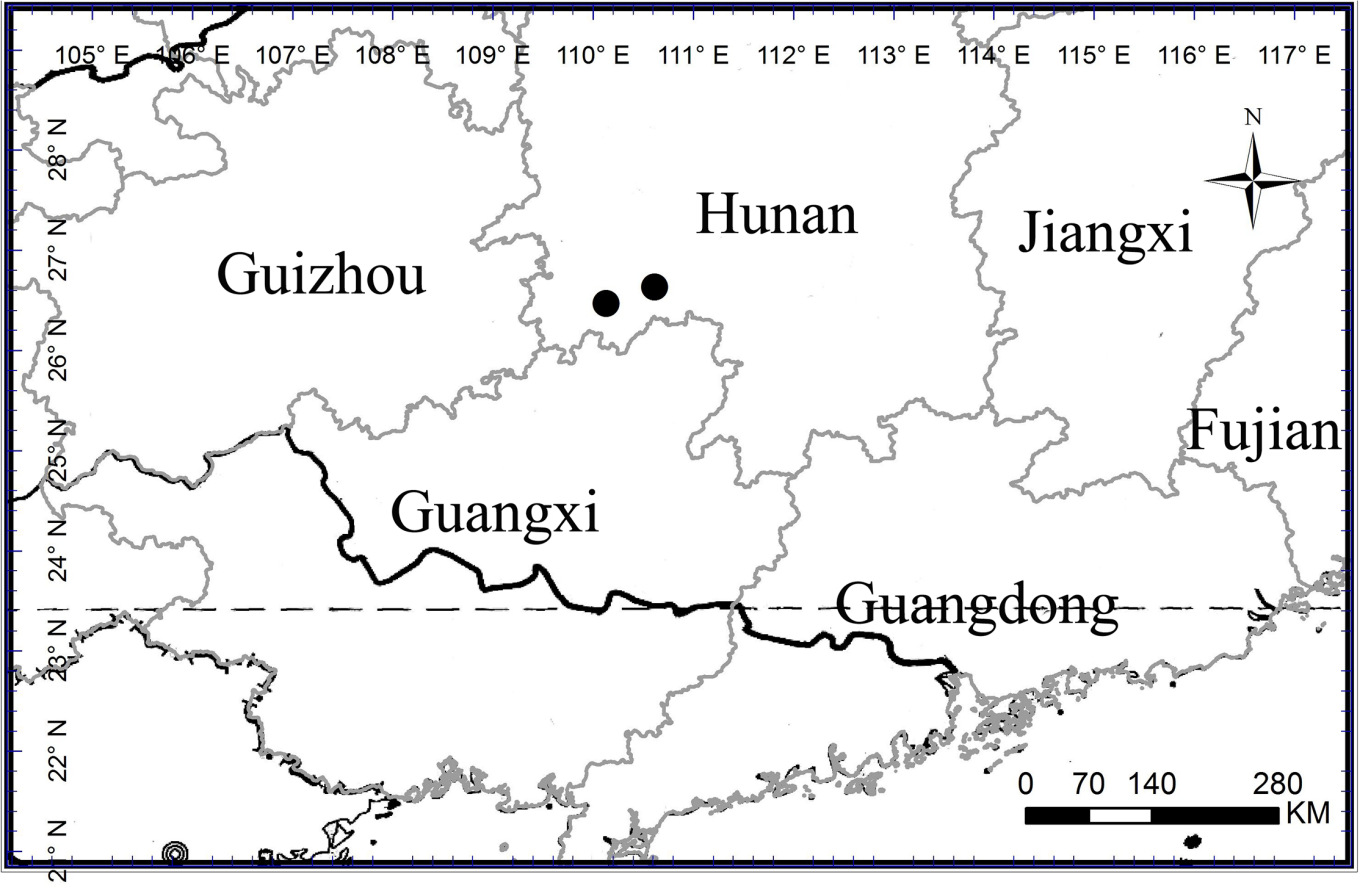
Distribution of *Lysimchia huangsangensis*.

*Lysimachia huangsangensis*is known from SW Hunan Province, China (Fig 3). It grows in scrubs or by the side of evergreen-deciduous and mixed-evergreen forest trails in association with *Acer davidii* Franch., *Bretschneidera sinensis* Hemsl., *Carpinus viminea* Lindl., *Cyclobalanopsis glauca* (Thunb.) Oerst., *C*. *sessilifolia* (Blume) Schottky, *Emmenopterys henryi* Oliv., *Camellia pitardii* Sohen-Stuart, *Maesa japonica* (Thunb.) Moritzi ex Zoll., *Symplocos ramosissima* Wall. ex G. Don, *Asarum caudigerum* Hance, *Carex cruciata* Wahl., *Impatiens hunanensis* Y.L. Chen, *Justicia quadrifaria* (Nees) T. Anderson, *Lysimachia congestiflora* Hemsl., *Miscanthus sinensis* Andersson and *Oplismenus undulatifolius* var. *japonicas* (Steud.) Koidz.

### Phenology

Found in flower from May to June and in fruit from September to October.

### Etymology

The epithet “*huangsangensis*” is derived from the type locality, Huangsang Natural Reserve, Suining Xian, Hunan Province, China.

### Additional specimens examined (Paratypes)

CHINA. Hunan: Wugang, Yunshan, ca. 950 m, 2 June 2014, *J*. *J*. *Zhou 140602001* (CSFI); the same locality, 19 October 2014, *J*. *J*. *Zhou 14101902 *(CSFI, IBSC).

### Conservation status

*Lysimachia huangsangensis* has only been collected in 2014 from two protected areas in southwest Hunan, China. However, it is not excluded that this species could be found in other localities and therefore it is classified as Data Deficient (DD) [60] according to the guidelines for using the IUCN Red List Criteria [61].

### Taxonomic position

According to the classification of Chen and Hu [8, 9], the new species belongs morphologically to Ser. *Drymarifoliae* Hand.-Mazz. [22]. Ser. *Drymarifoliae* can be divided into two groups based on calyx characters. The new species belongs to a group having crested calyx lobes together with *L*. *carinata*, *L*. *pterantha*, *L*. *baoxingensis*, *L*. *pteranthoides* and *L*. *crista-galli* (specimens examined are listed in S1 Text). *Lysimachia huangsangensis* is most similar to *L*. *carinata* and differs in the character of leaf blades, inflorescence and calyx-lobes. The new species can be distinguished from allied species by the following key.

1a. Leaf base cordate; crest of calyx lobes widest above middle.

2a. Leaves densely minutely bristly, crest of calyx lobes not forming a spur...... *L*. *pteranthoides*.

2b. Leaves strigillose or glabrescent, crest of calyx lobes forming a spur...................... *L*. *crista-galli*.

1b. Leaf base narrowly cuneate to rounded or truncate; crest of calyx lobes uniform in width or widest below middle.

3a. Leaves and calyx pubescent; crest of calyx lobes uniform in width........... *L*. *baoxingensis*.

3b. Leaves and calyx glabrescent; crest of calyx lobes widest below middle.

4a. Stems and pedicels pubescent......... *L*. *pterantha*.

4b. Stems and pedicels glabrescent.

5a. Calyx lobes linear-lanceolate, ca. 1 mm wide………………*L*. *carnata*.

5b. Calyx lobes ovate-lanceolate, 3–4 mm wide ………………*L*. *huangsangensis*.

### Phylogenetic relationship

**Fig 4:**
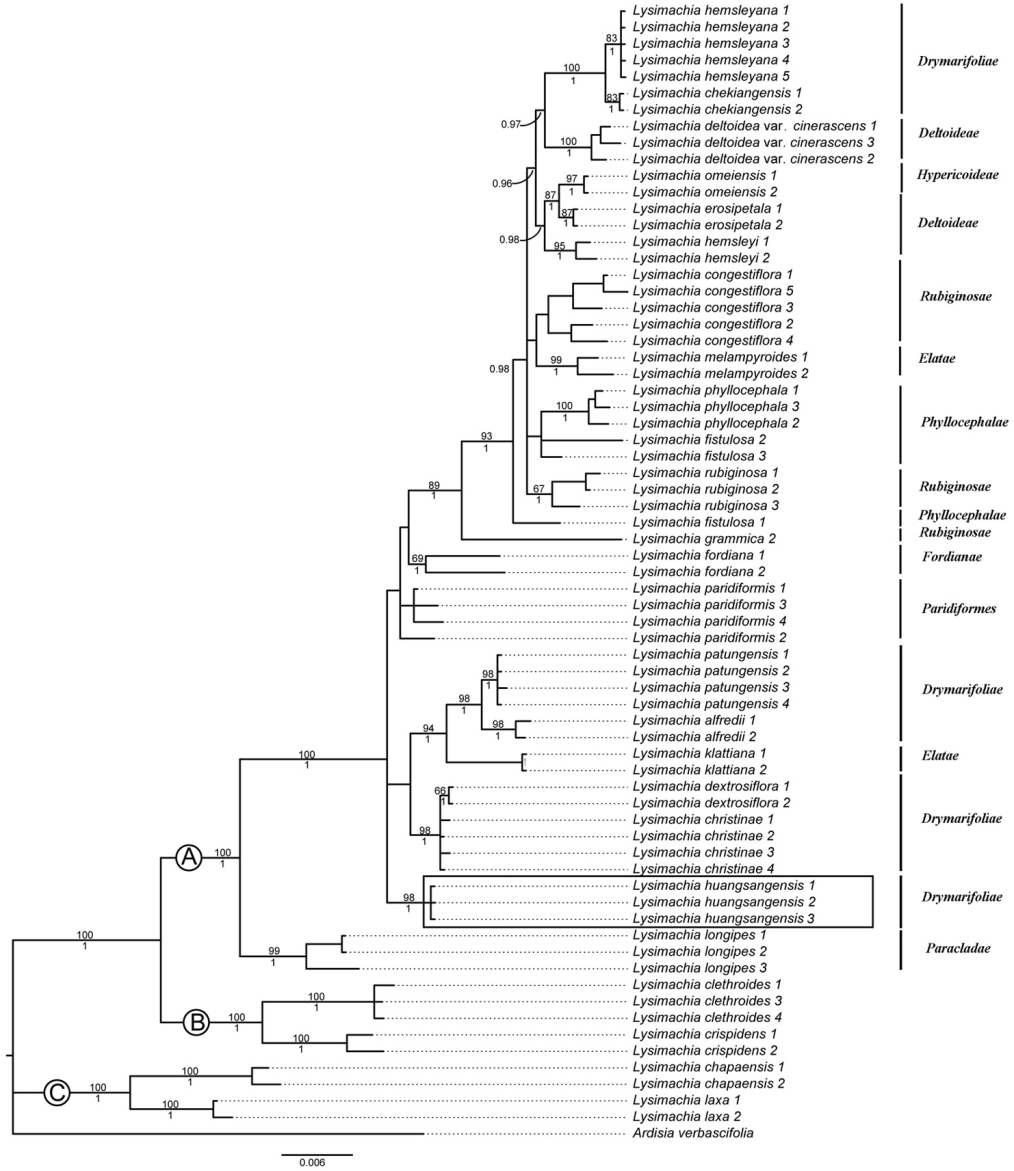
The Bayesian consensus tree of *Lysimachia* based on four molecular markers (*rbc*L, *mat*K, *psb*A-*trn*H, and ITS). The Bayesian posterior probabilities are shown below the branches and Maximum Likelihood (ML) bootstrap values above. Clade A, B, and C indicate subg. *Lysimachia* sect. *Hypericoideae*, subg. *Palladia* + subg. *Heterostylandra*, and subg. *Idiophyton* respectively. The boxed taxa represent the new species. Vertical bars with labels on the right side represent the series of sect. *Hypericoideae* proposed by Chen & Hu [8].

In the current study we conducted ML and BI analyses to determine the phylogenetic relationship of the new species. Both analyses generated congruent results and the Bayesian result is shown in Fig 4. The new species is a member of the Chinese sect. *Nummularia*, and forms a robust monophyletic group (Fig 4), though the phylogenetic position is not resolved.

## Discussion

In the classification of Chinese *Lysimachia*, Chen & Hu [8] divided the genus into five subgenera, i.e., *Idophyton*, *Lysimachia*, *Palladia*, *Heterostylandra* and *Naumburgia* on the basis of the classification of Handel-Mazzetti [22]. More than one third of the species are placed in subgen. *Lysimachia sensu* Handel-Mazzetti [22] and Chen & Hu [8], which is characterized by the yellow flowers that are solitary in the leaf axils or arranged in racemes, panicles or heads, the filaments forming a thin ring adnate to the corolla base, and the anthers opening with slits [5, 34]. The recent molecular evidence [34, 42, 43] reveals that subgen. *Lysimachia* is paraphyletic and consists of six groups. Sect. *Nummularia* is the largest section recognized in subgen. *Lysimachia* by Chen & Hu [8] and was further divided into ten series by them. Molecular studies [34] indicated that sect. *Nummularia* can be divided into two separate groups and Chinese taxa do not group with the type section. Chinese taxa of sect. *Nummularia* form a sister group with subgenera *Pallada* and *Lysimachiopsis* and differ from the latter by the flowers with oil-producing trichomes and filaments forming a thin ring adnate to the corolla base [5, 34]. Here, we treat Chinese taxa of sect. *Nummularia* as an independent section on the basis of the classification of Chen & Hu [8] and thus the name *Lysimachia* sect. *Hypericoideae* Knuth is the correct name for the section. The monophyly of sect. *Hypericoideae* has been supported by previous studies [34]. According to the subdivision of sect. *Hypericoideae* (as sect. *Nummularia*) by Chen & Hu [8] who further divided the section into ten series, the new species *L*. *huangsangensis* would be placed in ser. *Drimarifoliae* Hand.-Mazz. Our combined analysis (*rbc*L, *mat*K, *psb*A*-trn*H, and ITS) using Bayesian Inference (BI) (Fig 4) showed that the new species *Lysimachia huangsangensis* is nested in sect. *Hypericoideae*. Results support the placement of *L*. *huangsangensis* in sect. *Hypericoideae* and revealed that species in this section included two main groups, *L*. *longipes* representing ser. *Paracladae*, and other taxaof other series in this section. Our results do not support the classification by Chen & Hu [8]. Additional analyses using more molecular markers are necessary to clarify relationships within sect. *Hypericoideae*.

We delimited species by implementing the phylogenetic species concept [62–65]. In our phylogenetic analysis (Fig 4), *Lysimachia huangsangensis* forms a trichotomy together with other sampled taxa in sect. *Hypericoideae* except *L*. *longipes*. Although the phylogenetic relationships among sect. *Hypericoideae* are not well resolved, the recognition of *Lysimachia huangsangensis* is supported because all three accessions clearly cluster together. Morphologically, the new species can be distinguished from the closely related species by the leaf characters.

Pollen morphology and taxonomy of *Lysmiachia* was studies in several works [40, 66–73]. Amongst these Bennell & Hu [40] gave the most comprehensive account of the pollen morphology of *Lysimachia* and its taxonomic implications. They examined 98 species using light microscope, SEM and TM and ten major pollen types were recognized in relation to the infrageneric classification of Chen & Hu [8]. Pollen grains of sect. *Nummularia* including sect. *Hypericoideae* are medium-sized (>24–28 × 23–26) μm, subprolate (P/E<1.33), tricolporate, the tectum partial and coarsely reticulate [40]. In our observations, pollen grains of *L*. *huangsangensis* are medium-sized, subprolate, tricolporate, 29.39 (26.3–34.3) ×25.31 (23.2–27.1) μm, P/E = 1.36, and the tectum reticulate (Fig 2E and 2F). It supports the placement of the new species in Sect. *Nummularia sensu* Chen & Hu [8] or sect. *Hypericoideae*.

Surface features of seed coats are surprisingly little affected by the environmental conditions under which a plant grows [74]. Studies of seed morphology with SEM have revealed taxonomically useful microcharacters to support the delimitation of individual or groups of taxa [75]. Shao *et al* [76] observed microcharacters of seed surface of eleven Chinese *Lysimachia* species and divided them into two types, *Heterogenea-*type and *Grammica-*type, in relation to the subgeneric classification. Seven species of subgen. *Pallada* show the *Heterogenea-*type and four species of sect. *Hypericoideae* (as *Nummularia*in the sense of Chen & Hu [8]) show the *Grammica-*type with tuberculate ornamentation. Oh *et al*. [28] studied seed morphology and character evolution in the genus *Lysimachia* and its related genera (viz., *Anagallis*, *Ardisiandra*, *Asterolinon*, *Glaux*, *Pelletiera* and *Trientalis*). Seed morphology of 34 species of *Lysimachia* and 14 species of six related genera were investigated by them [28]. Three major types of seed shapewere identified, i.e., sectoroid (dorsiventrally and/or laterally flattened), polyhedral and coarsely rugose with a concave hilar area. The seed surface pattern of *Lysimachia* can be divided into six main types, i.e., reticulate, tuberculate, vesiculose, colliculate, undulate and poroid-alveolate. The seed morphological characters imply that the traditional concept of *Lysimachia* is not monophyletic and this was supported by the molecular studies [34, 41]. Oh *et al*. [28] observed seven species of sect. *Hypericoideae* (as subgenus *Lysimachia* F by them) and their results showed that the seed shape in sect. *Hypericoideae* is sectoroid or polyhedral with tuberculate seed surface pattern that is characterized by cells with convex epidermal facets. The observations of Oh *et al* [28] are similar to that of Shao *et al*. [76]. The seed of the new species *L*. *huangsangensis* is polyhedral in shape with side-faces slanted towards the ventral face (Fig 2G), outer layer of the surface with convex epidermal cells and the inner layer reticulate (Fig 2H). The seed morphology also supports the placement of the new species in sect. *Hypericoideae*.

## Supporting Information

S1 Table GenBank accession numbers for all the DNA sequences and voucher information. (XLS)

S1 Text The specimens of *Lysimachia carinata*, *L*. *pterantha*, *L*. *baoxingensis*, *L*. *pteranthoides* and *L*. *crista-galli* were examined to compare the new species with related species. (PDF)
