# Supplementary material for: Lysimachia huangsangensis (Primulaceae), a New Species from Hunan, China
Source: PLoS One. 2015 Jul 22;10(7):e0132713. doi: 10.1371/journal.pone.0132713 (PMC4511667; doi:10.1371/journal.pone.0132713)
Supplement: S1 Text — (PDF) [file pone.0132713.s002.pdf]

S2 Text 1. Specimens of its related species examined

Specimens of Lysimachia baoxingensis examined:–CHINA. Sichuan: Baoxing Xian, Dengchigou to Yemaoping, 2000 m, 28 September 1936, *K. L. Chu 3964* (IBSC, NAS); Baoxing Xian, Hedong, Yaodianzi, 1400 m, *T. H. Tu 4206* (PE); Jiulong Xian, Maoguchang to Wulaxi, 1600 m, 17 June 1979, *Z.A. Liu 20228* (IBSC); Luding Xian, Yaoxi Gongshe, Gonghe Dadui, 1800 m, 3 June 1980, *Q. Q. Wang 22569* (CDBI, IBSC); Luding Xian, Hailuogou, 1800 m, 9 July 2003, *G. Hao 455* (IBSC); Luding, Moxi, Hailuogou, 1600 m, 14 August 2007, *G. Hao 715* (IBSC); Ta-tsien-lou, Orleans s.n. (P); *Wilson 4019* (P).

Specimens of Lysimachia carinata examined:–CHINA. Guangxi: Yangshuo, 28 April 1938, *R. H. Shan 868* (NAS).

Specimens of Lysimachia crista-gallii examined:–CHINA. Chongqing: Fengjie Xian, Xinglong, Zhonghe Xiang, Dingxianxi, 28 June 1958, *Z. R. Zhang 25378* (IBSC).

Specimens of Lysimachia pterantha examined:–CHINA. Chongqing: Yangtze-Kiang, above Fee city, *Faber 332* (K); Wushan Xian, Wuxia, 150 m, May 1939, *T.P. Wang 10775* (WUK); Wushan Xian, near the city, 2 February 1958, *G. H. Yang 58221* (IBSC); Yunyang Xian, Chenjiagou, 25 May 1996, *Z. D. Chen et al. 960472* (PE).

Specimens of Lysimachia pteranthoides examined:–CHINA. Sichuan: inter Hualinpin et Lutingchiao, 1500 m, 2 July 1934, *Harry Smith 10337* (PE); Jinyang Xian, Heiquluo, 1960 m, 6 July 1960, *unknown collector 270* (IBSC); Miyi Xian, Malong Qu, 2100 m, 12 August 1959, *S. F. Zhu 20019* (PE). Yunnan: Tien-sin, 3000 m, E.E. Maire s.n. (P); Kiao Kia, 9 July 1908, F. Ducloux s.n. (P).
